# Early-Onset Euglycemic Diabetic Ketoacidosis Following Empagliflozin Initiation Triggered by Acute Influenza and a Ketogenic Diet

**DOI:** 10.7759/cureus.114227

**Published:** 2026-08-09

**Authors:** Madison S Meyer, Sushmithaa Ramesh, Yasmine Rammouni, Shoshanah Lasry, Hasan Sawan, Khalid Zakaria

**Affiliations:** 1 Medical Education, Wayne State University School of Medicine, Detroit, USA; 2 Internal Medicine, Henry Ford Providence Southfield Hospital, Southfield, USA; 3 Medical Education, Michigan State University College of Osteopathic Medicine, East Lansing, USA; 4 Medicine, Nova Southeastern University Dr. Kiran C. Patel College of Osteopathic Medicine, Davie, USA; 5 Medicine, Wayne State University School of Medicine, Detroit, USA; 6 Internal Medicine, Henry Ford Health, Novi, USA

**Keywords:** euglycemic diabetic ketoacidosis, influenza a, ketogenic diet, precipitating factors for euglycemic dka, sglt2 inhibitor-associated ketoacidosis

## Abstract

Euglycemic diabetic ketoacidosis (euDKA) is a form of diabetic ketoacidosis in which severe ketosis and acidemia occur despite mild hyperglycemia, leading to delayed diagnosis. EuDKA incidence has increased with widespread sodium-glucose cotransporter-2 inhibitor (SGLT2i) use, particularly during acute illness. While most reported cases occur after prolonged SGLT2i exposure, early-onset euDKA shortly after initiation remains rare. This case illustrates euDKA occurring within 48-72 hours of empagliflozin initiation in the setting of influenza A infection and a ketogenic diet, highlighting multifactorial euDKA pathogenesis and the need to reassess diabetes phenotype when insulin requirements are unexpectedly low. A 52-year-old female with type 2 diabetes mellitus presented with three days of flu-like symptoms, nausea, vomiting, dizziness, and poor oral intake. She initiated a ketogenic diet and empagliflozin 72 hours prior. Laboratory evaluation indicated high-anion-gap metabolic acidosis with a venous pH of 7.03, bicarbonate of 5 mmol/L, anion gap of 29, β-hydroxybutyrate of 7.0 mmol/L, and hyperglycemia (blood glucose 238 mg/dL). HbA1c was 10.6% (11.8% one month prior). She was admitted to the ICU and treated with fluids, insulin, and electrolyte monitoring. Despite severe acidemia, insulin requirements were minimal, totaling approximately 13.5 units, and she was transitioned to 5 units of glargine daily. Evaluation for autoimmune diabetes demonstrated preserved C-peptide (1.7 ng/mL) with negative glutamic acid decarboxylase-65 and islet cell antibodies. Adults presenting with ketoacidosis and low insulin requirements should prompt reassessment of diabetes phenotype when features are inconsistent with insulin-resistant type 2 diabetes. Continued SGLT2i therapy should be reconsidered to prevent recurrent metabolic decompensation.

## Introduction

Sodium-glucose cotransporter-2 inhibitors (SGLT2is) are a commonly prescribed class of oral medications for patients with type 2 diabetes mellitus (T2DM) because of their glycemic, cardiovascular, and renal benefits [1]. Despite these benefits, rare cases of euglycemic diabetic ketoacidosis (euDKA) have been reported in patients taking SGLT2i [2]. EuDKA can present with life-threatening metabolic acidosis and ketosis without profound hyperglycemia. Because this biochemical presentation differs from that of classic diabetic ketoacidosis (DKA), diagnosis and treatment may be delayed, leading to increased patient morbidity [3].

Patients with limited insulin reserve may be particularly vulnerable to euDKA when they begin taking SGLT2i medications. Latent autoimmune diabetes in adults is one cause of reduced β-cell reserve and is a subtype of autoimmune diabetes that typically occurs in adulthood and is frequently misdiagnosed as T2DM because of clinical features of apparent insulin independence and slow disease progression [4,5]. Although these patients are clinically thought to have T2DM, they continue to have gradual destruction of insulin-producing pancreatic β-cells and may have a predisposition to ketoacidosis, particularly during physiologic stressors such as acute infection [6]. Because adult-onset diabetes exists along a spectrum of autoimmune and non-autoimmune disease, assessment of diabetes-related autoantibodies may help clarify the underlying diabetes phenotype when clinical features are atypical [7].

Acute viral illnesses have been well-established precipitants of ketoacidosis [8]. Viral illnesses such as influenza trigger increased secretion of counter-regulatory hormones, dehydration, and relative insulin deficiency, promoting the risk of DKA [9]. If patients have minimal insulin reserve, ketoacidosis can occur after minor illnesses, even with only mild elevations in serum glucose [10].

This work was previously presented as a poster at the 2026 American Medical Association (AMA) Annual Poster Showcase on June 5, 2026.

## Case presentation

A 52-year-old female with poorly controlled T2DM presented to the ED following three days of progressive generalized weakness, fatigue, myalgias, chills, nausea, vomiting, exertional dyspnea, and markedly reduced oral intake after testing positive for influenza A. She tested positive for influenza A at an outpatient urgent care center 72 hours prior. She denied chest pain, abdominal pain, urinary symptoms, alcohol use, or any prior episodes of DKA. Her outpatient regimen consisted of 1,000 mg of metformin twice daily. Because of persistent hyperglycemia (HbA1c 11.8%), empagliflozin was added by her primary care physician three days before presentation.

On arrival at the ED, the patient was alert and oriented but appeared acutely ill. Vital signs revealed tachycardia with a heart rate of 124 beats per minute, blood pressure of 146/77 mmHg, respiratory rate of 16 breaths per minute, oxygen saturation of 100% on room air, and normothermia. Physical examination was notable for dry mucous membranes, clear lung fields, a benign abdominal examination, and no focal neurological deficits.

**Figure 1:**
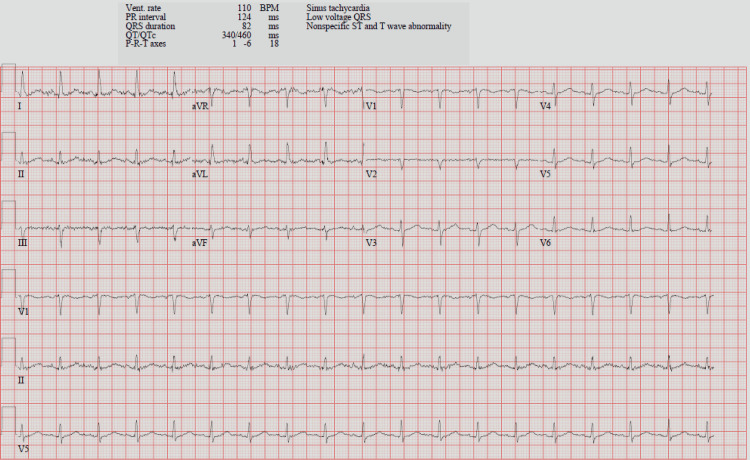
ECG on admission demonstrating sinus tachycardia without acute ischemic changes.

**Figure 2:**
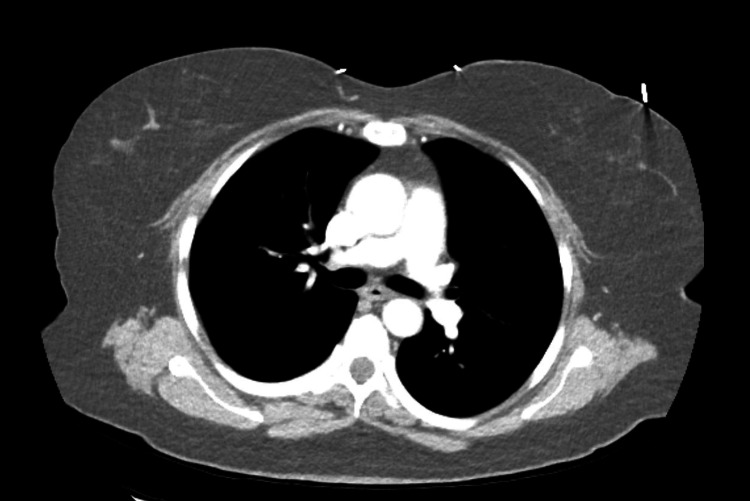
CT angiography of the chest demonstrating no evidence of pulmonary embolism.

Laboratory evaluation demonstrated marked leukocytosis (WBC 19.7 K/µL), severe high-anion-gap metabolic acidosis (anion gap up to 29), reduced serum bicarbonate (as low as 6 mmol/L), and profound acidemia on venous blood gas (pH 7.03). Beta-hydroxybutyrate was significantly elevated at 7 mmol/L. Serum glucose levels were only mildly elevated, ranging from 206 to 238 mg/dL, consistent with euDKA (Table 1). Serum lactate and renal function were normal. Urinalysis demonstrated prominent ketonuria and glucosuria without evidence of infection. The ECG showed sinus tachycardia without ischemic changes (Figure 1). CT angiography ruled out pulmonary embolism (Figure 2).

**Table 1:** Laboratory findings on admission.

Laboratory finding	Result (on admission)	Reference range
Glucose (serum)	238 mg/dL	70-110 mg/dL
Venous pH	7.03	7.35-7.45
Bicarbonate	5.8 mmol/L	22-29 mmol/L
Anion gap	29 mmol/L	8-12 mmol/L
pCO₂	22 mmHg	35-45 mmHg
Potassium	4.2 mmol/L	3.5-5.2 mmol/L
Urinalysis: leukocyte esterase	Negative	Negative
Urinalysis: nitrite	Negative	Negative
Urinalysis: WBC	2-5/hpf	0-5/hpf
Urinalysis: bacteria	Few	None seen
Urinalysis: squamous epithelial cells	0-5/hpf	0-5/hpf
HbA1c	10.60%	4.0-5.6%
Beta-hydroxybutyrate	7.0 mmol/L	<0.6 mmol/L

At this time, given the severity of the acidemia and metabolic abnormalities, the patient was admitted to the ICU for close monitoring. Empagliflozin was discontinued. The patient was treated with aggressive IV isotonic fluids and placed on a continuous IV insulin infusion per DKA protocol. Early initiation of dextrose-containing IV fluids was undertaken due to relatively normal serum glucose levels to allow continued insulin administration. The patient’s electrolytes were monitored closely throughout her stay, and she received potassium, phosphorus, and magnesium replacement during her course of treatment.

The patient was placed on oseltamivir for treatment of influenza infection, which had been started at an outside urgent care center prior to admission and was continued during the hospital course. The patient never required supplemental oxygen and remained hemodynamically stable throughout hospitalization. Serial ABGs showed gradual resolution, with sustained closure of the anion gap and resolution of acidemia over the course of 24-48 hours. She was transitioned to a subcutaneous insulin regimen, placed on a diabetic diet, and discharged from the ICU.

Subsequent workup revealed low-normal C-peptide levels with negative testing for autoimmune antibodies, including GAD65 and islet cell antibodies. Endocrinology recommended permanent discontinuation of SGLT2 inhibitor therapy and evaluation for autoimmune diabetes on an outpatient basis.

## Discussion

EuDKA presents a diagnostic challenge and is increasingly recognized as an adverse effect of SGLT2i treatment [11]. Unique to this case is the rapid development of euDKA within 48-72 hours of empagliflozin initiation, as well as the confluence of multiple risk factors leading to severe metabolic disturbance in a brief period of time. SGLT2i therapy promotes glycosuria and lowers plasma glucose levels independent of insulin [12]. The resulting reduction in circulating endogenous insulin and relative increase in glucagon activity drive both lipolysis and hepatic ketogenesis [13,14]. During physiologic stress such as acute viral illness, counter-regulatory hormone release further accelerates ketogenesis, while dehydration and decreased oral intake exacerbate relative insulin deficiency [2]. In this patient, acute influenza infection, ketogenic dieting, and recent SGLT2i initiation acted synergistically to create an environment favorable for rapid ketone accumulation despite only mild hyperglycemia.

An important feature of this case was the disproportionately low insulin requirement relative to typical weight-based DKA dosing. Despite severe acidemia, the patient required only approximately 13.5 units of insulin over the first 12 hours of treatment before transitioning to a low-dose basal insulin regimen, suggesting relatively modest insulin requirements despite profound metabolic derangement [9]. Although autoimmune markers were negative and C-peptide level was preserved, the clinical course prompted assessment of underlying β-cell reserve. C-peptide levels obtained during acute metabolic stress may not fully reflect functional insulin capacity, particularly in the setting of severe hyperglycemia or physiologic illness [15-17]. Furthermore, negative islet autoantibodies do not exclude reduced β-cell function, as adult-onset diabetes represents a heterogeneous spectrum that includes both autoimmune and non-autoimmune forms [7]. Rather than definitively establishing autoimmune diabetes, this presentation highlights the complexity of diabetes classification in adults and the need to consider limited insulin reserve even in patients labeled as having type 2 diabetes.

The patient’s ketogenic diet further compounded the risk. Very-low-carbohydrate diets promote physiologic ketosis and reduce insulin secretion [16]. When combined with SGLT2i therapy, this metabolic shift may lower the threshold for clinically significant ketoacidosis. This interaction emphasizes the importance of dietary counseling and reinforcement of sick-day management strategies when initiating SGLT2i therapy, particularly in patients with poor glycemic control.

## Conclusions

EuDKA remains a diagnostic challenge due to the absence of marked hyperglycemia, which can delay recognition and treatment. This case highlights the potential for rapid onset of severe euDKA within days of SGLT2i initiation, particularly in the presence of an acute viral illness, carbohydrate restriction, and reduced oral intake. Clinicians should maintain a high index of suspicion for euDKA in unwell patients receiving SGLT2i therapy.

In adults presenting with ketoacidosis and lower-than-expected insulin requirements, reassessment of diabetes phenotype and endogenous insulin reserve should be considered, even when autoimmune markers are negative. Early recognition and appropriate modification of therapy may improve diagnostic accuracy, guide long-term management, and prevent recurrent metabolic decompensation.
